# Peer Review Week 2021: a special thank you to our rapid communication reviewers

**DOI:** 10.2807/1560-7917.ES.2021.26.38.2109231

**Published:** 2021-09-23

**Authors:** Eurosurveillance editorial team

**Affiliations:** 1 European Centre for Disease Prevention and Control (ECDC), Stockholm, Sweden

On the occasion of Peer Review Week 2021, the *Eurosurveillance* editorial team would like to send out a warm thank you to all our Rapid communications reviewers (90 experts in 2021 so far). Rapid communications have been a special feature over the last 25 years, through which the journal has had an impact and served the public health and communicable disease communities with timely and authoritative evidence for rapid public health action. Our peer reviewers are essential in shaping the quality and usefulness of the published articles, especially on short notice and we acknowledge all those who have reviewed rapid communications for us in 2021, despite their high workload during the ongoing COVID-19 pandemic:

Cornelia Adlhoch; Marco Ajelli; Franz Allerberger; Erik Alm; Tommi Asikainen; Agoritsa Baka; Ian Barr; Senad Begić; Kaatje Bollaerts; Robert Booy; Maria José Borrego; Udo Buchholz; Saverio Caini; Jesus Castilla Catalan; Marco Cavaleri; Michelle Cole; Vittoria Colizza; Victor Corman; Laura Cornelissen; Benjamin Cowling; Natasha Crowcroft; Fortunato D'Ancona; Nicholas Davies; Brechje de Gier; Walter Demczuk; Aparna Dressler; Robert Dyrdak; Michael Edelstein; Dirk Eggink; Brendan Flannery; Arnaud Fontanet; Luca Freschi; Karthik Gangavarapu; Edward Goldstein; Thorolfur Gudnason; Eric Haas; Walter Haas; Thomas Harder; Kristin Hegstad; Derval Igoe; Michael G Ison; Michael Jackson; Kari Johansen; Annemarjut Jääskeläinen; Taishi Kayano; Marian Killip; Satu Kurkela; Amparo Larrauri; Kathy Leung; Bruno Lina; Michal Mandelboim; Andrea McCollum; Eleanor Mcnamara; Michael McNeil; Angeliki Melidou; Stefano Merler; Benjamin Meyer; Joël Mossong; Judith Mueller; Marcel Müller; Micha Nübling; Takis Panagiotopoulos; Maria Pardos de la Gandara; Pasi Penttinen; John Pettersson; Diamantis Plachouras; Katarina Prosenc; Giovanni Ravasi; Maximilian Riess; Julien Riou; Emmanuel Robesyn; Werner Ruppitsch; Gianpaolo Scalia-Tomba; Emily Scher; Gianfranco Spiteri; Carl Suetens; Jonathan Suk; Sheena Sullivan; Pham Thi Mui; Marianne van der Sande; Esther Van Kleef; Pauline Vetter; Qiang Wang; Matthijs Welkers; Eloise Williams; Thomas Williams; Emma Wiltshire; Shangxin Yang; Dominik Zenner; Walter Zingg.

We extend this acknowledgement to everyone who reviewed articles for us during the last 25 years and an excuse to those who reviewed a rapid communication this year and who we may have missed in this list.

